# Boerhaave syndrome, a rare oesophageal rupture: a case report

**DOI:** 10.29045/14784726.2021.3.5.4.49

**Published:** 2021-03-01

**Authors:** Rebecca Horrocks

**Affiliations:** North West Ambulance Service NHS Trust

**Keywords:** Boerhaave syndrome, oesophageal rupture

## Abstract

Boerhaave syndrome is a disorder mainly unknown among ambulance staff. However, the high mortality and morbidity rates associated with this rare disorder, and the fact that other conditions present with similar symptoms, suggest that this is one disorder to add to the differential diagnosis list. This case study describes a 17-year-old male complaining of left-sided ‘pressure’-type chest pain and persistent vomiting who on examination was found to have subcutaneous emphysema present. Deceived by a differential diagnosis, the patient was transferred under the belief that he had suffered a spontaneous pneumothorax as he was tall, young and thin. This case report reviews the literature surrounding Boerhaave syndrome and how it can present.

## Introduction

This case report will discuss an adult patient suffering from a ruptured oesophagus. This case has been chosen to be discussed due to the rarity of this condition, how it can often be misdiagnosed and how delays in diagnosis can lead to a poor outcome for the patient.

Oesophageal rupture, the transmural perforation of the oesophageal wall due to a rapid elevation in the intraluminal pressure of the oesophagus, is normally caused by healthcare interventions such as an endoscopy or oesophageal surgeries (Walls et al., 2018). Persistent and vigorous emesis in patients can lead to a lethal gastrointestinal tract disorder known as Boerhaave syndrome (BH): oesophageal rupture displaying symptoms of Mackler’s triad of chest pain, vomiting and subcutaneous emphysema (Turner & Turner, 2020).

With mortality rates of up to 40% due to BH, rapid recognition and surgical intervention is essential, though diagnosis of the disorder can be challenging (Aref et al., 2019). Clinicians can be plagued with differential diagnoses such as an aortic dissection, acute pancreatitis, myocardial infarction and a pnemothorax, when a patient who is suffering from BH requires quick diagnosis as survival rates of up to 75% have been recorded for those who are recognised and undergo surgery within 24 hours (Turner & Turner, 2020).

## Case presentation

An emergency ambulance crew, staffed with a senior paramedic (SP) and a newly qualified paramedic 1 (NQP1), were dispatched to a 17-year-old male complaining of chest pain and vomiting for approximately 24 hours. The first impression of the patient was that he was a tall, thin, young male in a tripod position with a pale complexion. The patient explained that he felt short of breath, had had left-sided chest pain (7/10 pain score) for approximately 24 hours, which felt like ‘pressure’ and was worse on inspiration, and he had vomited several times. He added that he could feel ‘bubbles popping’ in his neck. The patient had suffered from pneumonia a year earlier, no other medical history was noted and the patient did not take any regular medication. No food, fluid, bowel or urinary changes were noted, no family history was given and the patient had no known allergies. The patient stated that he smoked approximately five cigarettes daily and did not consume alcohol. The patient denied any chest trauma, though admitted that he had been boxing the night before.

An A-E (airway, breathing, circulation, disability, expose & examine) assessment followed. The crew ascertained that there were no danger or signs of catastrophic haemorrhage, and were able to begin a physical examination. The A-E survey determined any injury/illness that could be imminently life threatening (Caroline et al., 2014). Table 1 shows the A-E assessment.

**Table 1.** A-E assessment of patient.

Airway	Clear Open Self-maintained No tracheal deviation
Breathing	98% oxygen saturations (on room air) 24 respirations per minute Deep, laboured breathing with use of abdominal accessory muscles Bilateral air entry clear on auscultation – decreased air entry on left side, ‘flapping’ sound could be heard at the base of left lung Slight hyperinflation and hyperresonance on left side
Circulation	127 beats per minute pulse rate 126/87 mmHg blood pressure – sat down at time of reading 3 seconds capillary refill time Patient pale 12-lead ECG – sinus tachycardia No cyanosis Patient diaphoretic
Disability	5.8 mmol/L blood glucose level 36.2°C temperature Pupils equal and reactive to light, size 3 Glasgow Coma Scale 15
Expose and Examine	Subcutaneous emphysema present over sternocleidomastoid, and left side of the anterior chest No bruising, redness or open wounds noted

## Management/treatment

Based on the history and clinical assessment, with the presence of shortness of breath, chest pain, subcutaneous emphysema and the patient being a young, tall, thin male, the ambulance crew queried that the patient was suffering from a closed left-sided spontaneous pneumothorax.

The patient was transferred to the ambulance and sat in the High-Fowler’s position at his request to ease his respiratory effort, and this benefitted the NQP1 as she could see the patient clearly during transport. No supplementary oxygen was provided as the patient’s oxygen saturations were within a normal range. The patient’s increased respiration rate and deep laboured breathing mirrored the body’s defence mechanism to cope with the pneumothorax, which can make the situation worse (Leigh-Smith & Harris, 2005). The patient admitted that he felt very anxious, so the ambulance crew provided reassurance and coached the patient’s breathing to assist with slowing his respiration rate and to reduce the tachycardia.

An abnormal pulse and hypotension are recognised signs that a pneumothorax may be developing into a tension pneumothorax, which would require a needle decompression (Berry et al., 2019). Other signs and symptoms also include cyanosis, hypoxia, jugular vein distention and tracheal deviation (Leigh-Smith & Harris, 2005). Due to clinical concern that the patient may be at risk of developing a tension pneumothorax, the patient was monitored closely en route to the Emergency Department (ED) under emergency conditions with an amber standby passed to the receiving ED.

Intravenous (IV) access was gained and the patient was treated with IV paracetamol (1g) and IV Morphine Sulfate (10g) to treat his pain. Following pain relief, the patient’s respiration rate decreased to 20 per minute, the tachycardia slightly reduced to 116 per minute and the patient stated that his pain score had reduced to 5/10.

## Discussion

Reassurance was key for this patient; anxiety and panicking can exacerbate the symptoms of a pneumothorax, such as increased tachycardia, hyperventilating and clinical deterioration (Suarez et al., 2013). Through symptom analysis, relevant body system reviews and determining the relevance of events, the ambulance crew were able to come up with a plan for this patient. The key event to the call was the patient describing ‘bubbles popping’ in his neck, where the SP and NQP1 both came to the same conclusion of a closed pneumothorax, despite this not being the final diagnosis for this patient.

A pneumothorax, a thoracic disorder, is trapped air within the pleural space (Caroline et al., 2014). A primary spontaneous pneumothorax (PSP) is idiopathic in cause, whereas a secondary spontaneous pneumothorax occurs alongside the presence of a significant lung disease (Noppen, 2010). Choi (2014) analysed those diagnosed with PSP, concluding that tall, thin, young males aged between 10 and 30 years old were at an increased risk of PSP. Smoking was also recognised as a risk, as 91% of those diagnosed with PSP have declared themselves a current or past smoker. The findings of Tschopp et al. (2015) can be compared to this, where young, healthy people were found to be recurrently affected by a PSP, and where smoking has been established as the main risk factor. A review of data concerning hospital admissions for a spontaneous pneumothorax between 1968 and 2016 found that admission rates in England had increased from 9.1 to 14.1 per 100,000 population, with 73 per cent of these being male (Hallifax et al., 2018). Based on this, this patient was a high risk of a PSP. The crew had queried that this patient was suffering with a PSP based on his age, gender, smoking status, stature and the possibility of chest trauma from boxing the previous night.

Boerhaave syndrome, a gastrointestinal disorder, is recognised as the spontaneous rupture of the oesophageal wall (Tonolini & Bianco, 2013). First described by Herman Boerhaave, a Dutch anatomist and physician, in 1724 AD, following a post-mortem of a grand admiral who had developed severe left-sided chest pain following inducing vomiting after a feast, Boerhaave recognised the disorder as a very uncommon surgical emergency, and the term Boerhaave syndrome was born (Tamatey et al., 2013).

The most common cause of this disorder is thought to be vomiting, though other causes can include abdominal trauma, childbirth, defecation, epileptic seizures and weightlifting. The sudden increase in oesophageal pressure can lead to a trasmural tear through the oesophagus. Most commonly, rupture occurs in the left posterolateral wall of the oesophagus in the distal third of the area, which extends into the left pleural cavity causing damage to this area (Turner & Turner, 2020). The findings of Tamatey et al. (2013) outline how oesophageal perforations in the intrathoracic cavity can lead to gastric contents within this area, leading to emphysema, necrosis and medistinal inflammation

The occurrence of BH is still considered a rare presentation, with estimations of only 3.1 patients per 1,000,000 per year, and those considered to be most at risk are middle-aged males with a history of large food and alcohol consumption, though the patient population varies (Tonolini & Bianco, 2013). Signs and symptoms analysed by Tschopp et al. (2015) for clinicians to consider concerning this rare disorder include fever, lower thoracic pain, persistent vomiting, subcutaneous emphysema, tachycardia and tachypnoea. Based on this evidence, the patient had recognised symptoms of a rare BH presentation.

Differential diagnoses for BH include acute pancreatitis, aortic dissection, myocardial infarction and spontaneous pneumothorax, which can complicate identifying BH (Turner & Turner, 2020). The work of Ribeiro et al. (2018) outlined how persistent vomiting can lead to queries of acute pancreatitis, how left-sided chest pain radiating into the left shoulder can often lead to clinicians querying an aortic dissection or myocardial infarction, whereas subcutaneous emphysema can give a false impression of a pneumothorax, which all may deceive clinicians regarding the underlying BH.

The crew transferred the patient under emergency conditions, querying a PSP, and repeat observations were taken en route to the receiving ED to monitor for deterioration. Brown & Bleetman (2006) conducted a study in Birmingham concerning pre-alerts by ambulance crews, finding that 29 of the 52 critically ill patients did not have the relevant hospital staff awaiting the patient, as the crew had failed to pass a pre-alert. As there are no pre-hospital interventions for BH, having the relevant hospital staff present, and conveyance under emergency conditions, benefitted this patient by reducing the time taken for surgical intervention to take place.

The crew did not suspect BH, due to the rarity of the condition, though determined that the patient was acutely and severely unwell. Due to the presentation, suggestive of a thoracic disorder, as an ST elevation myocardial infarction had been ruled out with the use of a 12-lead ECG, the patient was advised that the transfer was being made based on the suspicion of a ‘collapsed lung’. The goal for this patient was to convey for further investigations and to gain a definitive diagnosis.

## Conclusion

The patient was assessed by the ambulance crew, who queried a PSP. The patient was treated with pain relief and monitored for deterioration en route to the receiving ED. History taking revealed persistent vomiting, which can be indicative of BH, though the crew were deceived by the patient’s age, gender, stature and subcutaneous emphysema, leading to them querying a PSP. Following hospital attendance, the patient was diagnosed with a rare case of BH and transferred to another hospital for surgical interventions.

Attendance at this case allowed the author to learn about a rare gastrointestinal complication, which can often be missed by clinicians. BH can be present as several other conditions, though late diagnosis and interventions can lead to increased mortality and morbidity rates. Despite querying the wrong condition, the crew’s treatment plan would remain unchanged, as there are no pre-hospital interventions in place for BH. Transport to hospital under emergency conditions with a pre-alert to the receiving ED benefitted this patient to reduce the time taken for transporting the patient for hospital investigations and interventions.

Case management of BH in the pre-hospital environment is largely unknown due to the rarity of the condition, and most medical literature is related to the hospital investigations and management of BH. A lack of research in the pre-hospital environment may also be due to how this condition presents with signs and symptoms similar to other conditions. The ambulance crew’s PSP diagnosis and management were justified based on this patient’s presentation, though adding BH as a potential differential diagnosis for these patients may benefit clinicians in the future.

## Guarantor statement

RH acts as the guarantor for this article.

## Conflict of interest

None declared.

## Funding

None.

## Informed consent

Verbal consent was gained from the patient to share his experience.
